# How Does Stroke Affect Skeletal Muscle? State of the Art and Rehabilitation Perspective

**DOI:** 10.3389/fneur.2021.797559

**Published:** 2021-12-23

**Authors:** Valentina Azzollini, Stefania Dalise, Carmelo Chisari

**Affiliations:** ^1^Department of Translational Research and New Technologies in Medicine and Surgery, DS Neurorehabilitation, University of Pisa, Pisa, Italy; ^2^Department of Neurorehabilitation, Pisa University Hospital - Azienda Ospedaliera Universitaria Pisana (AOUP), Pisa, Italy

**Keywords:** neurologic disorders, rehabilitation, chronic stroke, skeletal muscle, disability, long-term care

## Abstract

Long-term disability caused by stroke is largely due to an impairment of motor function. The functional consequences after stroke are caused by central nervous system adaptations and modifications, but also by the peripheral skeletal muscle changes. The nervous and muscular systems work together and are strictly dependent in their structure and function, through afferent and efferent communication pathways with a reciprocal “modulation.” Knowing how altered interaction between these two important systems can modify the intrinsic properties of muscle tissue is essential in finding the best rehabilitative therapeutic approach. Traditionally, the rehabilitation effort has been oriented toward the treatment of the central nervous system damage with a central approach, overlooking the muscle tissue. However, to ensure greater effectiveness of treatments, it should not be forgotten that muscle can also be a target in the rehabilitation process. The purpose of this review is to summarize the current knowledge about the skeletal muscle changes, directly or indirectly induced by stroke, focusing on the changes induced by the treatments most applied in stroke rehabilitation. The results of this review highlight changes in several muscular features, suggesting specific treatments based on biological knowledge; on the other hand, in standard rehabilitative practice, a realist muscle function evaluation is rarely carried out. We provide some recommendations to improve a comprehensive muscle investigation, a specific rehabilitation approach, and to draw research protocol to solve the remaining conflicting data. Even if a complete multilevel muscular evaluation requires a great effort by a multidisciplinary team to optimize motor recovery after stroke.

## Introduction

Stroke is the second leading cause of death worldwide (1), with chronic disability remaining in up to 50% of survivors (2). The improved acute care and the advances in early drug interventions after stroke, as the use of thrombolytic factors, have significantly increased the number of stroke survivors with a reduction of mortality (3). However, stroke survivors must cope with the long-term effects of stroke, suffering from persistent functional limitations that reduce autonomy in activities of daily life. Long-term disability caused by stroke is largely due to motor function impairment, and it is determined by primary and secondary changes to the acute event. The impairments can manifest progressively in the long-term, causing further modifications and adaptations: the lesions of descending neural pathways lead to altered neuromotor control and functional and structural changes of muscle tissue (4). Common symptoms in patients with stroke are weakness, hypotrophy, fatigability, and altered motor control, resulting from the combination of denervation, disuse, remodeling, and spasticity (5). Traditionally, the rehabilitation effort has been oriented toward the treatment of the central nervous system (CNS) damage with a central approach, which exploits the plastic capacity of the neural cells to recover the best motor control. In this paradigm, muscle tissue is often overlooked (6). In our opinion, to ensure greater effectiveness of rehabilitation, new protocol approaches are needed, focused on muscle modifications over time. The knowledge of the cascade of transformation at different scales—genetic, molecular, histological, biomechanical, morphological, neurophysiological, and clinical changes—could help to understand the temporality of the occurrence of these changes and prevent them. Here, we summarized the current knowledge on skeletal muscle changes and primary and secondary changes induced by stroke, looking for the actual literature evidence on specific rehabilitative treatments targeting the muscle. Our idea is that only a “multi-target” rehabilitation treatment, which considers both the periphery and the CNS, can improve outcomes for stroke survivors until the best possible recovery. Based on this aim, we analyzed the current knowledge on modification of muscle in stroke, considering three fundamental aspects: morphology, metabolism, and electro-mechanical properties. For each of them, we resumed the principal treatment proposals available in the literature.

## Morphological Changes

Morphological changes may occur in parallel at a different level, including intrinsic fiber muscle phenomena (loss of muscle mass, muscle thickness, and decrease in the physiological cross-section area), but also at a more general tissue rearrangement, referring to sarcomere shortening and histological changes in the extracellular matrix. These two phenomena may mutually influence each other's and be hardly individualized, but for a clearer and more identifiable discussion they will be treated differently in this review. In the first section, we described the “stroke-induced sarcopenia,” referring to muscle atrophy as a specific intrinsic fiber muscle phenomenon, and in the second section, we refer to “stiffness” as general tissue rearrangement. Furthermore, it should be mentioned that in several neurological disorders, characterized by spastic paresis, a dedicated term is used to qualify the specific muscle contracture (spastic myopathy), characterized by both increased muscle tension and stretch-sensitive evolution (7).

### Stroke-Induced Sarcopenia

In healthy people, muscle tissue is gradually lost during aging, resulting in a decrease in mass and strength, a condition described as sarcopenia (8). In specific pathological conditions, especially in those that may invoke inflammatory processes, disease-related immobility or malnutrition, sarcopenia can occur as secondary, defining a “specific sarcopenia” (6). Recently, the muscle atrophy, consequent to the stroke has been defined as a new condition called “stroke-induced sarcopenia” or “stroke-related sarcopenia”; it has also been associated with worse clinical outcomes and physical dysfunction (6, 9). Furthermore, the impaired function predisposes stroke survivors to inactivity that might contribute to deconditioning, fatigue, and further functional loss (10, 11). Stroke-induced sarcopenia arises from the combination of multiple mechanisms, including immobilization and dysfunctional atrophy, impaired feeding, inflammation, sympathetic overactivation, and denervation (6). The prevalence of stroke-related sarcopenia is higher than the one of general population, matching age, gender, and race of healthy individuals, indicating a specific pathway (12). Furthermore, this prevalence during the first month is 50% and it is ~34% after 6 months (13), suggesting that the adaptive responses in muscle tissue may be most pronounced early after stroke (5). Moreover, the loss of muscle mass after stroke is commonly accompanied by fat deposition, often associated with a common stroke risk factor such as obesity, worsening the outcome (14). These early changes in muscle, such as loss of muscle mass, reduced fiber cross-sectional area (CSA), and increased intramuscular fat deposition, occur between 3 weeks and 6 months after stroke in both paretic and non-paretic limbs (15–18). In a recent study it was shown that the presence of sarcopenia, associated or not with obesity (sarcopenic-obesity), affects the improvement of activities of daily living (ADL), dysphagia, and discharge rates to home, so the researchers stated that the treatment of stroke-induced sarcopenia in a rehabilitative setting is crucial (19). The same researchers also demonstrated that skeletal muscle mass gain at the end of rehabilitative treatment is significantly associated with improved functional outcomes in patients with sarcopenia after stroke (20).

Some researchers suggested that sarcopenia could be due to the activation of catabolic pathways, especially the ubiquitin–proteasome system (UPS), autophagy, and apoptosis (21, 22). These mechanisms seem to be in part due to the downregulation of the gene of Sirtuin1 (SIRT1), a key regulatory factor of the energetic status of the cell, counteracting metabolic and age-related disorders. Reduced expression of the SIRT1 gene and reduced SIRT1 activity, resulting in skeletal muscle atrophy, have been demonstrated in post-stroke animal models; similarly, attenuation of muscle atrophy was found when SIRT1 is overexpressed (23). Moreover, it was demonstrated that the expression levels of myostatin mRNA, which downregulate the skeletal muscle growth, are 40% higher in the paretic than non-paretic muscles in stroke survivors (24). This could be due to intramuscular fat accumulation, consequent to stroke, that may cause insulin resistance, and the subsequent hyperinsulinemia has been shown to increase serum myostatin (14). The knowledge of the molecular mechanisms of stroke-induced sarcopenia allows to identify specific therapeutic approaches. In fact, in a pre-clinical study, the use of resveratrol (RESV), an exercise mimetic drug, during the early acute phase of stroke, limited muscular atrophy, through the activation of SIRT1, and normalized the hypertrophy of slow-twitch muscle fibers (I, IIa), suggesting that RESV may improve oxidative metabolism in stroke-affected muscles (25). In humans, it is well-known that exercise is the most effective method for sarcopenia treatment (6). Indeed, a recent study highlighted that resistive training for 12-weeks (3 times/week) could reduce the myostatin mRNA expression levels and stimulates significant muscle hypertrophy and intramuscular fat reductions (24). Furthermore, it is known that a stroke patient could suffer from malnutrition, and its early recognition or misdiagnosis significantly affects the outcomes; in fact, malnutrition aggravates sarcopenia because muscle and adipose tissue wasting occurs (26, 27). In this regard, it becomes essential to evaluate the nutritional status and provide nutritional management in association with specific exercise interventions, to ensure the best result; therefore, as described in the literature, the combination of exercise and nutritional therapy is the most appropriate choice to obtain a positive effect on the increase of skeletal muscle mass (20, 26, 27).

Finally, in a very recent study, it was highlighted that muscles of the trunk undergo atrophy in a later post-stroke phase, causing a worsening of the balance (28); this suggests the need to accurately plan both the type of rehabilitation and the timing: patients should be treated pointing on different aims at different stroke phases, for example, in addition to continuous motor control training over time, strengthening and endurance training of trunk muscles could be helpful during the chronic phase.

### Stiffness

Paresis induced by stroke leads to a reduced active voluntary movement and a reduced joint range of motion (ROM), often associated with an active or passive mobilization hyper-resistance or stiffness.

Stiffness can be due to “neural” or “non-neural” phenomena, such as spasticity, spastic dystonia, or respectively, muscle contractures and soft-tissue fibrosis (29).

Consequently, long-term secondary complications, such as soft tissue contractures, pain, pressure sores, decreased ADL, social isolation resulting in decreased quality of life, can occur (30, 31). These phenomena seem to be linked to structural changes of intrinsic muscle properties over time, with an increase of intramuscular connective tissue and fat content (32). It has been shown that these changes depend on the deposition of hyaluronan in the extra-cellular matrix (ECM) (33), intramuscular fat (34), and lead to increased viscosity and could immobilize muscle in the shortened position (29). Indeed, fibrosis and abnormal accumulation of materials in the ECM leads to an increased collagen content and an altered orientation of collagen that likely contributes to a greater transverse tensile stiffness against radial expansion and fascicle shortening. There are several approaches to stiffness management (35). An early passive motion and stretching are the most used techniques to prevent and treat muscle shortening. In particular, it was demonstrated that fixed muscle length induces muscle atrophy, by activation of specific gene expression (36). However, a recent Cochrane systematic review concluded that stretching procedures performed for 3 months or less do not improve joint mobility (37), even if in a recent study 1 year of stretching was found to be effective in counteracting muscle architecture modification (fascicle length, thickness) among plantar flexors, linked with a clinical meaningful gain in ankle dorsiflexion angle (38). This suggests that, in clinical chronic conditions, stretch interventions may need to be continued for longer than just a few weeks (29).

Stiffness in the muscle will generate a constant stimulation of its spindles, triggering the activation of Ia fibers, further tension, failure to release contraction, and a muscle change as serious as the hyperactivity severity (spasticity) (39). When stiffness is linked to spasticity or spastic dystonia the more effective and safe, recommended treatment in a rehabilitation setting is the inoculation of Botulinum Toxin Type A (BoNT-A) (40). BoNT-A blocks the release of acetylcholine at the neuromuscular junction, producing a chemical denervation, with the aim of reducing excessive muscle activity without producing significant functional weakness (41). Unfortunately, the effect of BoNT-A serial injections in muscle, largely used to treat spasticity and spastic dystonia in stroke, is poorly investigated, although in an animal model research, a noxious BoNTA-related effect on muscle has been suggested (42); and in humans, the histological recovery of muscle seems to remain incomplete after the BonTA injection (43). To avoid this potentially damaging effect, a tempestive treatment, integrated with early stretching treatment, could be a proper strategy. Indeed, even if BoNT-A injections are the gold standard in patients with spasticity in the chronic phase of stroke (44), recent studies showed that BoNT-A treatment performed within 3 months since stroke onset in naïve patients with spasticity can achieve the maximum effect on muscle tone, allowing better control of spasticity in chronic phase and a reduction of serial injections (45–47). Moreover, a careful systematic follow-up of treated patients is necessary (48).

Resuming, stiffness is a complex phenomenon consisting of the “reflex-” and “nonreflex-mediated” resistance to passive movement, so each of these needs to be quantified uniquely and related with its corresponding clinical facets. An appropriate treatment of stiffness should consider both aspects, for example, different adjuvant rehabilitation treatments could be useful to reduce soft tissue contracture (“nonreflex-mediated” resistance, rheological aspect), and combined with BoNT-A injection could boost its effect on “reflex-mediated resistance” (49); in fact, allowing a decrease of muscle overactivity may not be sufficient to obtain morphological changes such as an increase in sarcomeres or fascicle length. In addition, an adequate and time-dependent treatment could be designed, also regarding any unresponsive patients in which alternative treatments (e.g., surgical therapy) should be considered.

## Muscular Metabolism Changes

Stroke patients exhibit impaired metabolism compared to healthy subjects, with increased tissue lactate and glycerol production, delayed and impaired glucose utilization, and slightly increased energy expenditure (4). This contrasts with the “normal” age-related sarcopenia process, where there is a shift from fast-twitch type IIa/b to slow-twitch type I fibers, which is mainly attributed to the disuse of fast fibers (50, 51). The paretic muscle of chronic stroke patients shows a smaller overall fiber cross-sectional area (CSA) with a shift toward a low oxidative type IIX fiber content and a reduced type I and type IIA fiber content (52). As a consequence, muscle resistance in affected limbs is likely decreased, because type IIX fibers are more prone to fatigue, leading to impaired muscle performance (50). Acute stroke patients rely on carbohydrate utilization during prolonged walking, while healthy individuals rely mostly on fatty acids oxidation; this carbohydrate utilization likely indicates preferentially anaerobic metabolism and potentially limits the ability to walk for a long time (53). Conversely, in the chronic phase, other researchers did not find differences in skeletal muscle tissue substrate metabolism between paretic and not paretic leg, even if the energy consumption seems to be higher in the not paretic one; in these studies the increased glycolytic activity and reduced lipolytic activity in post-stroke skeletal muscle suggest a bilateral shift in fiber type (54, 55). However, it is noteworthy that in some specific conditions, such as sarcopenic obesity post-stroke (mentioned above), fast-type muscle fibers switch to slow-type muscle fibers resulting in decreased muscle mass and strength, as in age-related sarcopenia (14). Although these data seem to be in contrast, they actually highlight the importance of careful characterization of a patient's muscular metabolism through a specific evaluation over time. In a rehabilitative view, focusing on muscles could identify ways to reverse stroke-induced metabolic alteration; indeed, different therapeutic physical interventions (i.e., aerobic exercise or neuromuscular electrical stimulation) could exert different biological effects and can improve physical performance (56). High-intensity training, aimed to increase type IIA fiber percentages, might contribute to muscle power and endurance, crucial for functional capacity (52, 57). Aerobic exercise normalized the CSA of type I and IIb muscle fiber and increased peroxisome proliferator-activated receptor gamma coactivator 1-α (PGC1α) protein content, which is indicative of increased aerobic capacity (25). To date, these contradictions are not completely clarified and become more evident in the analysis of motor unit activity changes, as discussed in the Electromechanical changes section. Research efforts should be aimed at resolving these apparent contradictions, correlating them with the time course of the chronicity of stroke, and the first step could be to reposition the main movement effector, the muscle, in the crosshairs.

## Electromechanical Changes

Impaired voluntary muscle motion is also caused by a change in the motor unit (MU) activation. In the first phase post-stroke (in 4 h), there is an initial reduction of MU number with a larger amplitude of the outlier surface of MU action potential (MUAP) and a decrease in compound muscle action potential (CMAP) amplitude, that could continue for a long time (58–60). It may be related to the trans-synaptic inhibition of the spinal alpha-motor neurons, as a result of upper motor neuron involvement (58, 61). Despite this, in the chronic phase, it was reported that the number of MUs increased, particularly in patients with mild stroke (62); in the researchers opinion, this suggests that the initial decrease of MUs is due to functional inactivity and, therefore, its recovery is realistic over time (62, 63). Regarding the larger MUAPs amplitude described in chronic stroke patients, it could indicate enlarged MU, possibly due to reinnervation (collateral sprouting) (64). Furthermore, normal recruitment order, based on the size of the MUs, is also altered in the muscles of the paretic limb: the recruitment of larger MUs at higher muscle contraction levels is less evident in the paretic muscles than in the contralateral ones. Additionally, the threshold strength range for MU recruitment has been compressed to a lower level on the affected side, indicating a different type of MU fiber (65), with a hypertrophy of slow-twitch skeletal muscle fibers and an atrophy of fast-twitch fibers (66). It is confirmed by the study of muscle fiber conduction velocity (MFCV). The MFCV is inferior in the paretic than in the non-paretic muscles of stroke patients (67). Similarly, this might indicate an increase in the proportion of type I fibers compared to type II (68). The same pathophysiology observed in the paretic limb may be present in the non-paretic limb, although to a lesser degree, due to the presence of the ipsilateral corticospinal tract in humans; indeed, in some individuals, up to 30% of corticospinal axons may descend in the ipsilateral ventral tract (58, 69). Nevertheless, the reduction in MUs' number correlates with the reduced muscle mass in paretic limbs, but not in the non-paretic one, suggesting other factors for the reduced muscle mass in these patients as described above (58). Using conventional surface EMG, different parameters (such as power spectrum, spike distribution, clustering index) were described to be different in hemiparetic muscle and healthy ones, and for some of these different hypotheses were suggested, including central and peripheral process (increased motor unit synchronization, impairments in motor unit control properties, loss of large motor units, and atrophy of muscle fibers) (70–72).

Other information could be provided from the analysis of electromechanical delay (EMD), which represents the time elapsed from the onset of active state in skeletal muscle (onset of signal to surface electromyogram—sEMG), and the onset of voluntary strength development. Indeed, a recent study found that the EMD is longer in the paretic than in the non-paretic triceps surae muscles. The longer EMD on the paretic side may be associated with the reduced torque-generating capacity, and it could be linked to the modified intrinsic properties of muscle tissues. The elongation of the EMD is likely attributable to electrochemical processes (i.e., altered ion conductance at neuromuscular junction level, alterations in the excitation–contraction coupling mechanism, disturbed propagation of MUAPs), but also, with a predominant role, to mechanical processes. In particular, the longer EMD on the paretic side may potentially support the hypothesis of a fast-to-slow fiber shift. Moreover, longer EMD is also associated with higher shear wave speed, an elastography ultrasound biomarker of muscle stiffness; so, a stiffer muscle may potentially lead to longer EMD, since a greater muscle activation may be needed to deform muscle shape and to shorten fascicles to generate an adequate strength in a stiffer paretic muscle (73). While the biopsy and metabolism studies results suggest a prevalence of type 2 fiber in stroke muscles, the increased activation of low-threshold MUs and the EMD elongation indicate a relative increase in type 1 MU. These discrepancies could be explained by several hypotheses. First, often different studies focused their attention on different muscles (large muscles of limbs vs. muscles of the hand); second, sometimes the samples are very heterogeneous with patients at different post-stroke times. So, it is possible that the two alterations coexist and that the underlying mechanisms occur in different post-stroke phases. It might be interesting to draw a longitudinal study by monitoring muscle changes with different methods (metabolic and electrophysiological) to better understand how and when they occur, allowing to manage a specific and timely rehabilitation intervention aimed at recovery.

From a rehabilitative point of view, there is a positive correlation between the root mean square of MUAP and Fugl–Meyer score, which indicates a relation between MU properties and clinically assessed motor recovery (64). In this sense, the altered MU activation might be a target of rehabilitative treatment: it is shown that a rehabilitative treatment provided by robotic-assisted locomotion system (Lokomat) induces a significant increase in firing rate, not accompanied by an increase in strength; this could suggest an effect of training on motoneuronal firing rate that thus contributes to muscle motor control (74, 75).

## Limitations

For the preparation of this review, we did not apply the methodology of a systematic review with subsequent limitations. Systematically traceable search criteria are not represented and PRISMA criteria have not been considered. Furthermore, we focused on the muscle properties trying to separate this from motor control disorder, so some factors of muscle weakness (i.e., recruitment and firing rate) are only partially mentioned from a rehabilitative point of view. Despite the growing number of publications, the data still show some conflicting findings and methodological limitations, probably due to the lack of patient stratification based on correct functional assessment.

## Conclusions and Recommendations

From this review, it is clear that changed descending neural output, caused by stroke, leads to functional and structural changes in skeletal muscle. The main muscle changes are morphological modifications, alteration in muscular metabolism, and electromechanical features. For each of these aspects, specific treatment approaches (medical or rehabilitative) are present in several preclinical studies, showing effectiveness in improving motor function and ability to modify the muscular features recovering the “normal.” Despite this, the same approach in clinical studies is not equally carried out. Indeed, also in specific rehabilitative settings, a realistic evaluation of muscle function is not performed yet: often the only muscle evaluation is limited to subjective functional assessment measure without any attention to the morphological, metabolic, and electro-mechanical properties of muscle. In our opinion, the focus onto the muscle means to consider that the impaired motor function in stroke is the result of both reduced central motor control activity and muscle modifications. In this perspective, for optimal stroke management, it would be necessary to investigate the two components simultaneously, investigating the alterations of the descending motor pathways using techniques such as transcranial magnetic stimulation and motor-evoked potentials, and verifying the peripheral muscle modifications, not only with a clinical assessment, but also with an instrumental evaluation through ultrasound techniques, bioimpedance analysis, or more sophisticated metabolic and gene analyses. A second aim of our review was to focus on the need for specific muscular rehabilitation protocol in clinical practice, suggesting an integrated approach for the best motor recovery. To achieve this ideal management, research must aim to solving the contradictions still present in skeletal muscle modifications, by better investigating the muscle during the different phases of stroke, from the onset to the chronic rehabilitation phase. Therefore, it would be appropriate to design a specific longitudinal study in which muscle changes are investigated using different methods to define the time course of the specific variations and to manage a specific and timely rehabilitation intervention aimed at recovery. In particular, it must be clear that at different stages it would be necessary to modulate and change the type and the aim of the treatment. This ambitious goal must be positioned as an outcome of a multidisciplinary intervention, in which several experts work together to integrate the bottom-up with the classic top-down paradigm with a “multi-target” rehabilitation approach.

Based on the current literature available, we can make the following recommendations for optimal stroke management:

- Muscle assessment in stroke patient should be performed together with CNS evaluation, to address a specific patient-tailored rehabilitation.
- Muscle assessment should be conducted through a multi-level screening:

- Morphological evaluation: ultrasound techniques, bioimpedance analysis, elastography, or magnetic resonance.
- Metabolic change should be directly investigated through immunohistochemical analysis or indirectly by dosing metabolic substrates.
- Electromechanical changes (EMG and sEMG).

- Nutritional screening might be performed to assess energy requirements and provide adequate integration for muscle mass gain.
- Designs of specific treatment protocols based on the modifications highlighted (aerobic training, resistance training, stretching, or medical therapy for stiffness).
- The treatment should be time-dependently designed.
- Verify treatment effect at muscle level over time (longitudinal study).
- Multidisciplinary approach is strongly recommended.

## Author Contributions

VA and SD: conceptualization, writing, draft preparation, and writing the original article. VA wrote the manuscript with support from SD. SD and CC: provided critical feedback and helped shape the manuscript. All authors contributed to the final version of the manuscript and approved the submitted version.

## Conflict of Interest

The authors declare that the research was conducted in the absence of any commercial or financial relationships that could be construed as a potential conflict of interest.

## Publisher's Note

All claims expressed in this article are solely those of the authors and do not necessarily represent those of their affiliated organizations, or those of the publisher, the editors and the reviewers. Any product that may be evaluated in this article, or claim that may be made by its manufacturer, is not guaranteed or endorsed by the publisher.
